# Supplementary material for: Video-based detection of Delirium in hospitalized adults
Source: PLOS Digit Health. 2026 May 29;5(5):e0001462. doi: 10.1371/journal.pdig.0001462 (PMC13221075; doi:10.1371/journal.pdig.0001462)
Supplement: S1 Text — Supplementary Methods. (DOCX) [file pdig.0001462.s001.docx]

## Supplementary Methods

**Deep Learning Model Training and Testing Specifications**

DeepLabCut was utilized to fine-tune the ResNet101 model for human pose estimation on the patient video dataset. The input image size was 1920x1440 pixels, and the output was 35 pairs of coordinates corresponding to each keypoint. The algorithm was fine-tuned for 700,000 iterations with default parameters.

Training Parameters

- Training Fraction: 0.8 (Proportion of dataset split into training)
- dot_size: 3 (point size)
- batch_size: 8
- optimizer: sgd (stochastic gradient descent)
- lr_init: 0.0005 (initial learning rate)
- max_iters: 789,000 (maximum iterations)
- multi_step: (learning rate schedule)
  - [0.005, 10000]
  - [0.02, 430000]
  - [0.002, 730000]
  - [0.001, 1030000]
- init_weights: resnet101 (initial weights)
- scale_jitter_lo: 0.5
- scale_jitter_up: 1.25 (each image was rescaled within range of jitter values to augment training)
- p-cutoff: 0.812 (likelihood threshold for visibility)

The algorithm was evaluated on a held-out testing dataset, comprising of 20% of the total dataset. Model performance was assessed using Euclidean distance for human labeled points that were identified by the model with a sufficient confidence threshold. Euclidean distance was defined as $\sqrt{{(x_{m}-x_{g})}^{2}+{(y_{m}-y_{g})}^{2}}$ where $(x_{g},y_{g})$ are the ground truth labels and $(x_{m},y_{m})$ are the machine-labeled points. The confidence threshold for the model (p-cutoff) was selected to optimize the F1 score, which is the harmonic mean of precision and recall of point detection on the training dataset.

**Deep Learning Model Benchmarking**

The fine-tuned model termed “DLC” was evaluated against FaceMesh and BlazePose from the MediaPipe suite. The FaceMesh and BlazePose models were trained on datasets of 30k mobile camera photos of people-in-the-wild and 85k photos of people performing common poses or fitness exercises, respectively. These models were used as is and not fine-tuned on our video cohort. Model performance was compared by using 33 shared points that were identified by FaceMesh, BlazePose, and DLC. The conversion between DLC, BlazePose, and FaceMesh points is detailed in the data_key.csv file.

**Feature Pre-Processing and Selection**

Given the large number of behavioral features calculated based on the video data, feature selection was performed to improve model fit. In addition to using all features, different feature selection methods were considered: chi-square based univariate selection, lasso regression, mutual information (MI), and minimum redundancy maximum relevancy (mRMR) [27]. Additionally, the number of features selected (k) was varied between 10, 20, 40, and 80 for the chi-square, MI, and mRMR-based methods.

For chi-square based feature selection, the feature data was min-max scaled to ensure it was non-negative before performing feature selection using the SelectKBest function in scikit-learn. Lasso regression was implemented on the feature data using the LassoCV linear model where features with non-zero coefficients were selected. mRMR was implemented using the mrmr-selection package. Mutual Information was implemented using the *mutual_info* function from scikit-learn.

**Delirium Classification Models**

Four different machine learning models were fit: support vector machine, logistic regression, extreme gradient boosting [26], and random forest-based models. Support vector machine, Logistic regression, and the Random Forest model were implemented using the *LinearSVC*, *LogisticRegression,* and *RandomForestClassifier* functions from the sci-kit learn library, respectively. The Gradient Boosting model was implemented using the *XGBClassifier* function from the xgboost library.

**Missing Values**

Behavioral features with missing values were imputed using zero substitution. Behavioral features with infinite values, which can arise from ratios when the denominator approaches zero, were replaced with the maximum or minimum finite value observed in that feature across the training dataset. This approach was applied uniformly prior to any feature selection or model fitting.

**Considerations for Future Implementation**

The quality of video data (lighting, occlusion, etc.) and its effect on model predictions was not formally evaluated. Future work should define protocols for handling poor quality input video data. User interaction is required in three phases: video capture, video screening, and interpretation of model results. The camera should be mounted at the foot of the hospital bed as described in Methods: Video Data Collection. Prior to being used in the deep learning model, the videos were manually screened to ensure they did not contain fully masked patients, head wraps from epilepsy monitoring, or other video file errors. Future research could automate this process of screening videos. The output of the model is intended to serve as a screening tool rather than a standalone clinical diagnosis and should be interpreted in the context of clinical findings by a trained healthcare professional.
