# Supplementary figures and images for: Video-based detection of Delirium in hospitalized adults
Source: PLOS Digit Health. 2026 May 29;5(5):e0001462. doi: 10.1371/journal.pdig.0001462 (PMC13221075; doi:10.1371/journal.pdig.0001462)

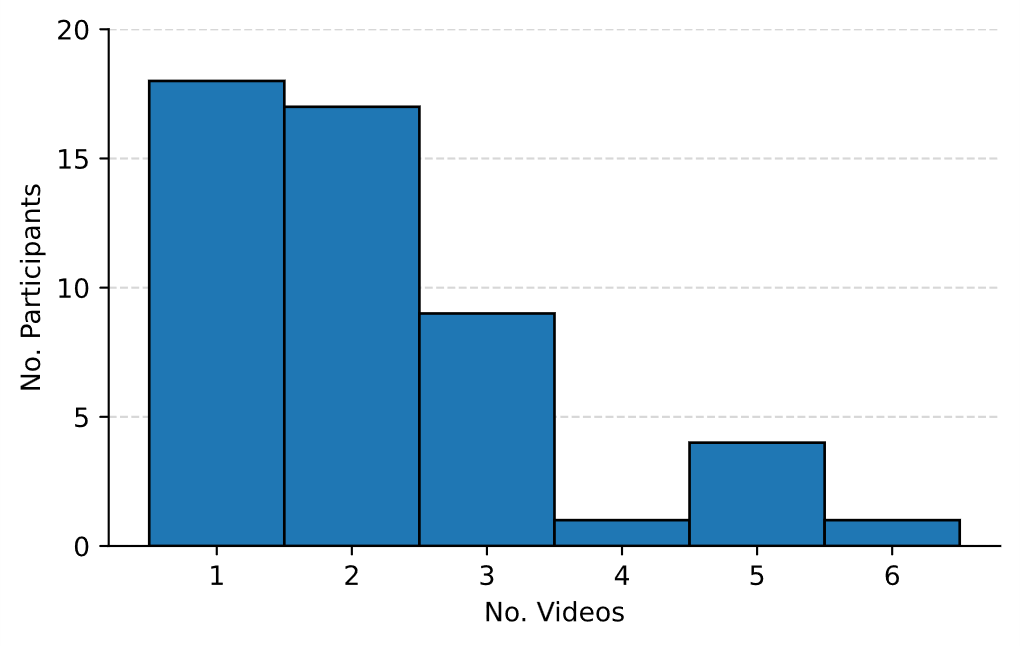

Supplement: S2 Fig — Assessment of repeated measures, showing how many participants contributed each number of videos per participant in the comprehensive dataset. (DOCX) [file pdig.0001462.s004.docx]

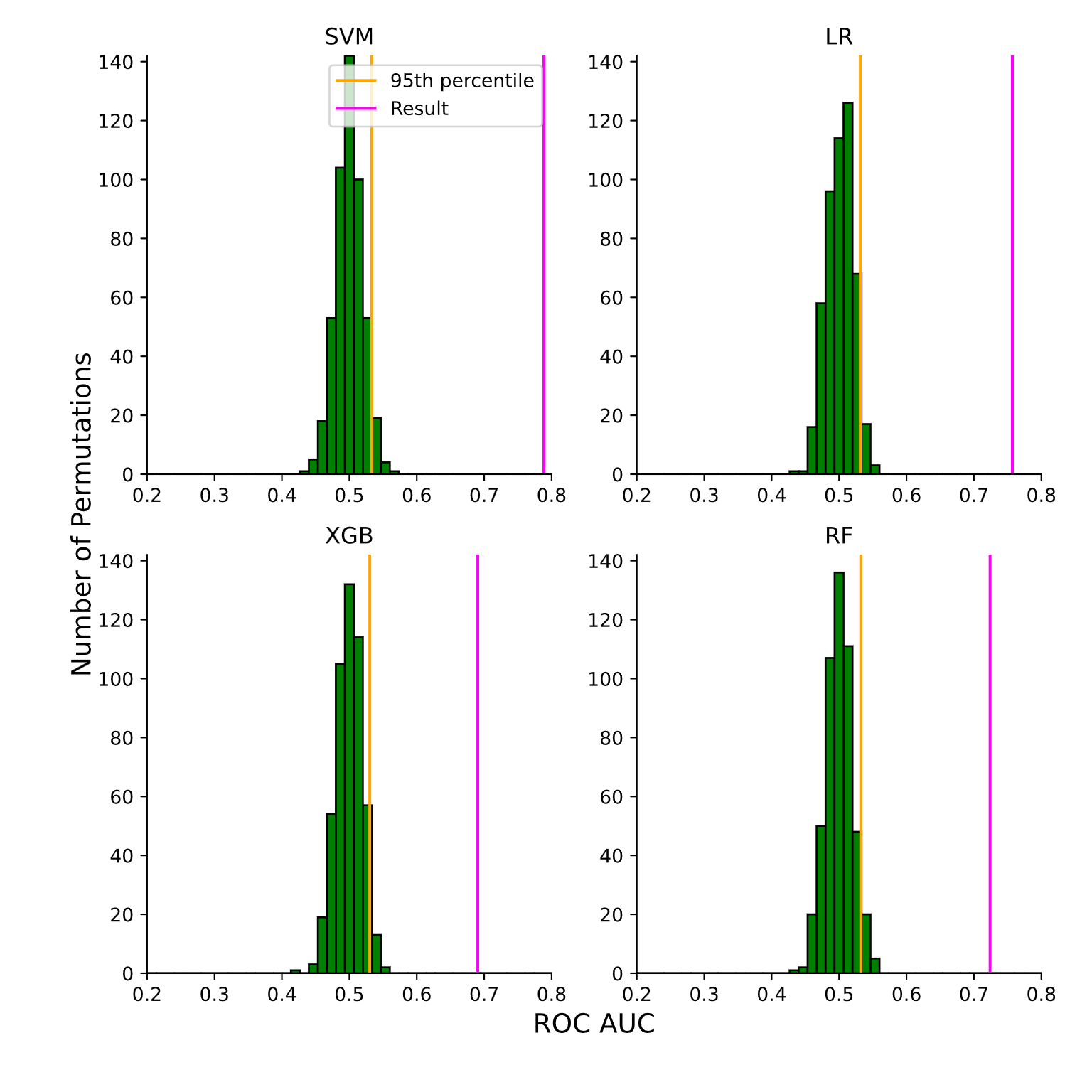

Supplement: S3 Fig — Permutation Analyses for Different Machine Learning Models. Models were fitted on a shuffled version of the video dataset for 500 trials using the same feature selection and model fitting procedure. The 95th percentile of the null distribution is denoted by the orange line. Estimated model performance is plotted using the pink line for support vector machine (SVM), logistic regression (LR), extreme gradient boosting (XGB), and random forest (RF) algorithms. (DOCX) [file pdig.0001462.s005.docx]

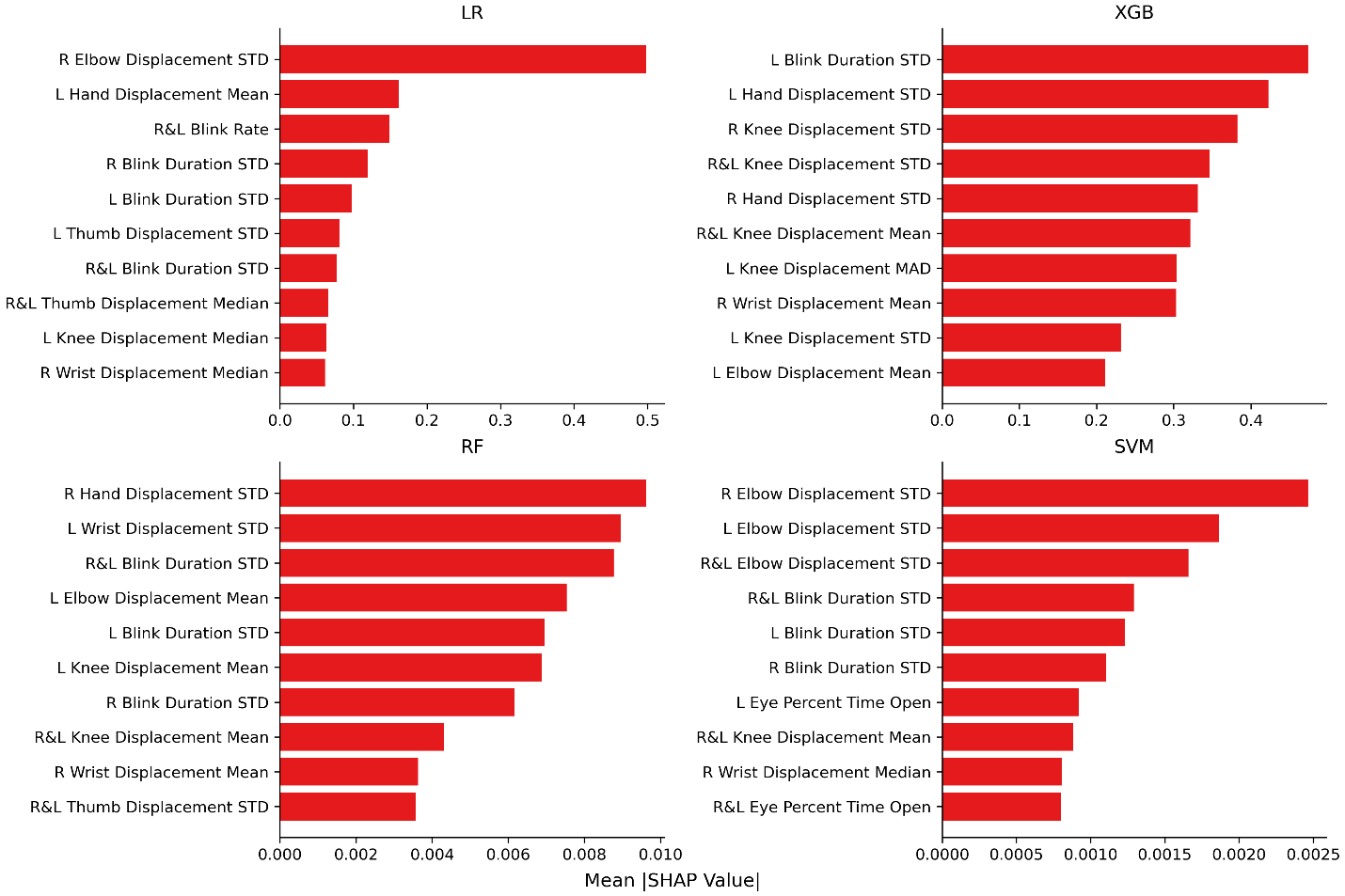

Supplement: S4 Fig — Feature Importance for Different Machine Learning Models. The top 10 features used in each algorithm are ranked in descending order of importance by their mean absolute Shapley analysis (SHAP) values across folds and repetitions for the best combination of algorithm and feature selection method. Features of the highest importance clustered around the upper extremities and eyes. Displacement was measured as the frame-by-frame change in position for the keypoints, with summary measures being taken across all changes between frames. “R” denotes right, “L” denotes left, and “R&L” denotes that the metric was averaged across both sides to calculate this feature. “STD” denotes standard deviation, while “MAD” denotes mean absolute deviation. See S4 Table for further descriptions of how each feature was calculated. (DOCX) [file pdig.0001462.s006.docx]
