# Supplementary material for: Video-based detection of Delirium in hospitalized adults
Source: PLOS Digit Health. 2026 May 29;5(5):e0001462. doi: 10.1371/journal.pdig.0001462 (PMC13221075; doi:10.1371/journal.pdig.0001462)
Supplement: S2 Table — Composition of Restricted and Comprehensive Model Datasets. The Total column represents the total numbers of labeled frames in the Initial Restricted or Subsequent Comprehensive datasets. Frames were split into Training and Testing sets for the Comprehensive dataset. The Restricted dataset also included a Validation set with videos not included in Training or Testing sets. (DOCX) [file pdig.0001462.s008.docx]

**Initial/Restricted Model Dataset:**

|  | **Total** | **Training** | **Testing** | **Validation** |
| --- | --- | --- | --- | --- |
| # frames | 400 | 251 | 63 | 86 |
| # videos | 45 | 35 | 25 | 10 |
| # patients | 40 | 35 | 25 | 10 |

**Comprehensive Model Dataset:**

|  | **Total** | **Training** | **Testing** |
| --- | --- | --- | --- |
| # frames | 782 | 625 | 157 |
| # videos | 109 | 109 | 81 |
| # patients | 50 | 50 | 44 |
