# Supplementary material for: Video-based detection of Delirium in hospitalized adults
Source: PLOS Digit Health. 2026 May 29;5(5):e0001462. doi: 10.1371/journal.pdig.0001462 (PMC13221075; doi:10.1371/journal.pdig.0001462)
Supplement: S3 Table — Video Feature Domains. (DOCX) [file pdig.0001462.s009.docx]

| **Domain** | **Features** | **Number** |
| --- | --- | --- |
| Eye movement | Pupil movements, horizontal pupil movements, vertical pupil movements, horizontal eyelid axis, vertical eyelid axis | 15 |
| Blinks | Blink rate, blink duration, eye aspect ratio, % of frames with eyes open | 7 |
| Head movements | Roll, pitch, yaw, and nose aspect ratio | 4 |
| Mouth movements | % of frames with mouth open, mouth vertical distance, mouth horizontal distance, upper lip movement, and lower lip movement | 6 |
| Upper extremity | % of frames with hands higher than elbow, % of frames with hands higher than chin, wrist displacement, hand displacement, elbow movement, shoulder movement, arm movements (wrist-shoulder-elbow angle) | 23 |
| Lower extremity | Knee movement | 3 |
| Summary Statistics | mean, median, standard deviation, and median absolute deviation | X 4 |
| Total |  | 232 |
