# Supplementary material for: Video-based detection of Delirium in hospitalized adults
Source: PLOS Digit Health. 2026 May 29;5(5):e0001462. doi: 10.1371/journal.pdig.0001462 (PMC13221075; doi:10.1371/journal.pdig.0001462)
Supplement: S4 Table — Behavioral Feature Descriptions. Each row defines a group of related behavioral kinematic features considered as inputs for the delirium classification algorithm, how they were calculated, and any references. Variable names follow the convention _ < extremity > _ < laterality> for unilateral measures (e.g., right or left), or avg_ < summary statistic > _ < extremity> for bilateral measures representing the average of the right and left sides. Summary statistics include the mean, median, standard deviation (std), and mean absolute deviation (MAD). (DOCX) [file pdig.0001462.s010.docx]

| **Feature Names** | **Variable Names** | **Calculation** | **Notes** |
| --- | --- | --- | --- |
| Percent eyes open, Percent right eye open, Percent left eye open | - perc_eye_open - R_eyes_open - L_eyes_open | % of frames where at least one pupil is detected, % of frames where right pupil is detected, % of frames where left pupil is detected |  |
| Percent mouth open | - per_mouth_open | % of frames where the mouth vertical distance was greater than the mouth horizontal distance times .3 |  |
| Mean upper lip movement, mean lower lip movement, average mean lip movement, median upper lip movement, median lower lip movement, average median lip movement, upper lip movement variability, lower lip movement variability, average lip movement variability, upper lip movement median absolute deviation, lower lip movement median absolute deviation, average lip movement median absolute deviation | - mean_Tmouthdiff - mean_Bmouthdiff - Avg_mean_mouthdiff - median_Tmouthdiff - median_Bmouthdiff - Avg_median_mouthdiff - std_Tmouthdiff - std_Bmouthdiff - Avg_std_mouthdiff - MAD_Tmouthdiff - MAD_Bmouthdiff - avg_MAD_mouthdiff | Upper lip distance was calculated as the difference in position between consecutive frames, lower lip distance was calculated as the difference in position between consecutive frames |  |
| Mean right pupil movement, mean left pupil movement, etc. | - mean_Rpupildist, - mean_Lpupildist, - Avg_mean_pupildist - median_Rpupildist - median_Lpupildist - Avg_median_pupildist - std_Rpupildist - std_Lpupildist - Avg_std_pupildist - MAD_Rpupildist | Right pupil movement was calculated as the distance between consecutive frames, left pupil movement was calculated as the distance between consecutive frames |  |
| Mean right vertical pupil movements, mean left vertical pupil movements, etc. | - mean_RVeyemvmt, - mean_LVeyemvmt, - avg_mean_Veyemvmt, - median_RVeyemvmt, - median_LVeyemvmt, - avg_median_Veyemvmt, - std_RVeyemvmt, - std_LVeyemvmt, - avg_std_Veyemvmt, - MAD_RVeyemvmt, - MAD_LVeyemvmt, - avg_MAD_Veyemvmt | 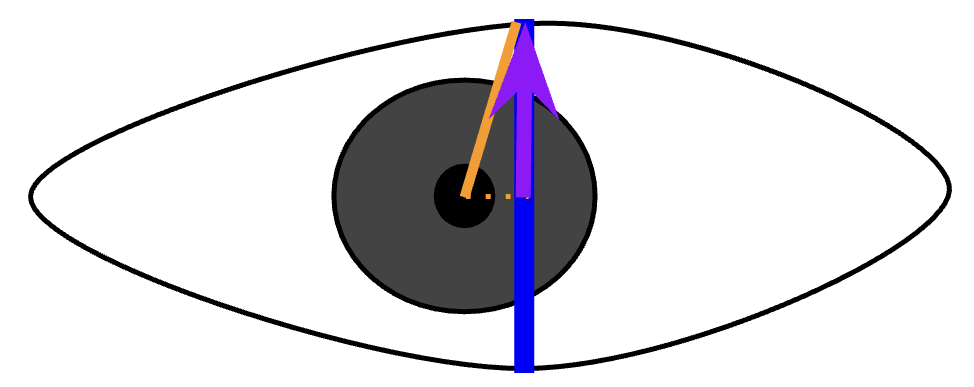  $\frac{pupilR-eyelidTop \cdot vertical\_eyelid\_axis}{\parallel vertical\_eyelid\_axis\parallel}$  Right vertical pupil movement is represented by the purple vector |  |
| Mean right horizontal pupil movement, mean left horizontal pupil movements, etc. | - mean_RHeyemvmt, - mean_LHeyemvmt, - avg_mean_Heyemvmt, - median_RHeyemvmt - median_LHeyemvmt, - avg_median_Heyemvmt, - std_RHeyemvmt, - std_LHeyemvmt, - avg_std_Heyemvmt, - MAD_RHeyemvmt, - MAD_LHeyemvmt, - avg_MAD_Heyemvmt | 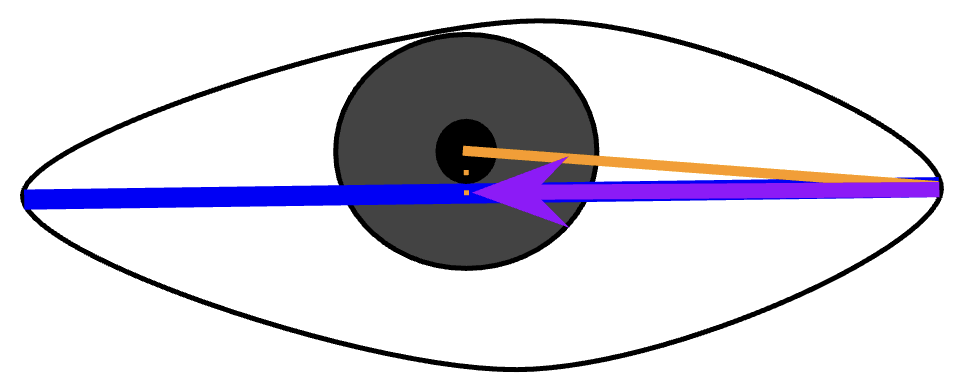  $\frac{pupilL-eyelidmed \cdot horizontal\_eyelid\_axis}{\parallel horizontal\_eyelid\_axis\parallel}$  Left horizontal pupil movement is represented by the purple vector |  |
| Mean right horizontal eyelid axis length, mean left horizontal eyelid axis length, etc. | - mean_RHeyelidaxis, - mean_LHeyelidaxis, - avg_mean_Heyelidaxis, - median_RHeyelidaxis, - median_LHeyelidaxis, - avg_median_Heyelidaxis, - std_RHeyelidaxis, - std_LHeyelidaxis, - avg_std_Heyelidaxis, - MAD_RHeyelidaxis, - MAD_LHeyelidaxis, - avg_MAD_Heyelidaxis | 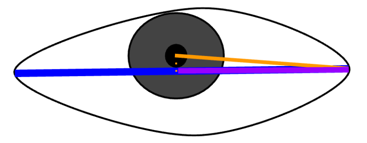  The horizontal right eyelid axis is represented by the blue vector |  |
| Mean right vertical eyelid axis length, mean left vertical eyelid axis length, etc. | - mean_RVeyelidaxis - mean_LVeyelidaxis - avg_mean_Veyelidaxis - median_RVeyelidaxis - median_LVeyelidaxis - avg_median_Veyelidaxis - std_RVeyelidaxis - std_LVeyelidaxis - avg_std_Veyelidaxis - MAD_RVeyelidaxis - MAD_LVeyelidaxis - avg_MAD_Veyelidaxis | 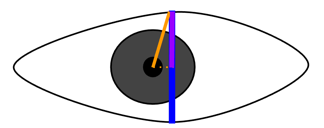  The vertical right eyelid axis is represented by the blue vector |  |
| Mean right eye aspect ratio, mean left eye aspect ratio, etc. | - mean_R_EAR - mean_L_EAR - avg_mean_EAR - median_R_EAR - median_L_EAR - avg_median_EAR - std_R_EAR - std_L_EAR - avg_std_EAR - MAD_R_EAR - MAD_L_EAR - avg_MAD_EAR | 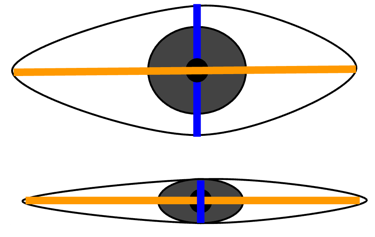    Eye aspect ratio helps gauge eye openness and is calculated as the ratio of vertical eyelid distance and horizontal eyelid distance.  $\frac{\parallel vertical\_eyelid\_axis\parallel}{\parallel horizontal\_eyelid\_axis\parallel}$ | Rosebrock, A. (2017, April 24). Eye blink detection with OpenCV, Python, and dlib. *Pyimagesearch*. <https://pyimagesearch.com/2017/04/24/eye-blink-detection-opencv-python-dlib/> |
| Mean vertical mouth distance, mean horizontal mouth distance, etc. | - mean_Vmouthdist - mean_Hmouthdist - avg_mean_mouthdist - median_Vmouthdist - median_Hmouthdist - avg_median_mouthdist - std_Vmouthdist - std_Hmouthdist - avg_std_mouthdist - MAD_Vmouthdist - MAD_Hmouthdist - avg_MAD_mouthdist | Vertical mouth distance is calculated as the distance between the upper and lower lip points. Horizontal mouth distance is calculated as the distance between the left and right mouth points. |  |
| Mean right blink duration, mean left blink duration, average blink duration, etc. | - mean_Rblinkt - mean_Lblinkt - avg_mean_blinkt - median_Rblinkt - median_Lblinkt - avg_median_blinkt - std_Rblinkt - std_Lblinkt - avg_std_blinkt - MAD_Rblinkt - MAD_Lblinkt - avg_MAD_blinkt | Blink duration is calculated by examining the number of frames between eyes open-to-close and close-to-open events. Eyes were defined as close when the pupil was not detected. |  |
| Right eye blink rate, left eye blink rate, average blink rate | - Rblink_rate - Lblink_rate - avg_blink_rate | Blink rate is calculated as the number of blinks per minute. Blinks are defined based on pupil detection. Blink rate was specifically equal to the number of eyes open-to-close events divided by the frame rate and multiple by 60. |  |
| Percent of frames where the right hand is higher than the right elbow, percent of frames where the left hand is higher than the left elbow, percent of frames where the right hand is higher than the chin, percent of frames where the left hand is higher than the chin | - Rhandhigher_elb - Lhandhigher_elb, - Rhandhigher_chin - Lhandhigher_chin | The % of frames where the hand is higher than the elbow is calculated by comparing the y coordinate of the hand and elbow points in frames where both were detected. This is a rough proxy for carphology.  The % of frames where the hand is higher than the chin is calculated by comparing the y coordinate of the hand and chin points in frames where both were detected. This is a rough proxy for carphology. |  |
| Roll | - mean_roll - median_roll - std_roll - MAD_roll | Roll head pose captures rotation in-plane movements. Roll was calculated as $\arctan\left( \frac{y_{1}}{x_{1}} \right)$  where y_1_ is the difference in the y coordinates between the left and right eye midpoints and x_1_ is the difference in the x coordinates between the left and right eye midpoints. Roll angle is an “estimate of the inclination angle on the roll axis” and is defined with respect to current eye position here (Arcoverde et al., 2014)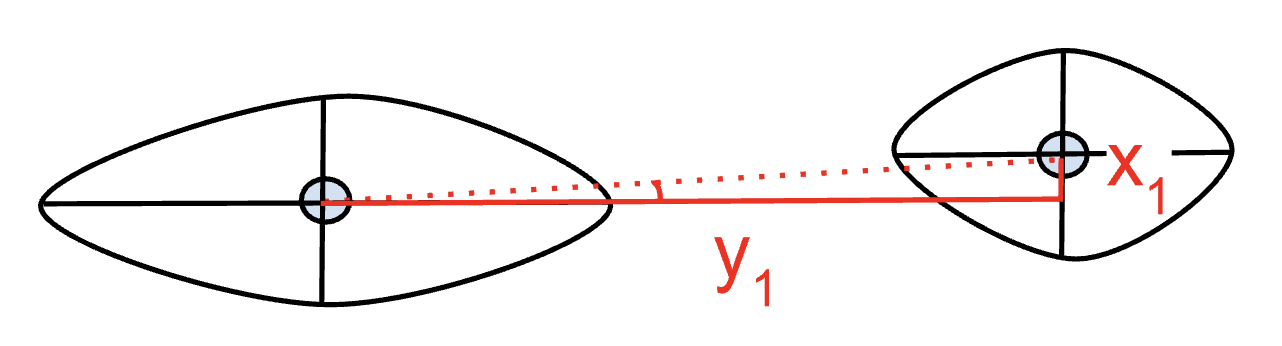 | Arcoverde Neto, E. N., Duarte, R. M., Barreto, R. M., Magalhães, J. P., Bastos, C. C. M., Ren, T. I., & Cavalcanti, G. D. C. (2014). Enhanced real-time head pose estimation system for mobile device. *Integrated Computer-Aided Engineering*, *21*(3), 281–293. <https://doi.org/10.3233/ICA-140462> |
| Pitch | - mean_pitch - median_pitch - std_pitch - MAD_pitch | Pitch head pose captures head up and down movements. Pitch was calculated as the differences in the nose y coordinates between consecutive frames. | Arcoverde Neto, E. N., Duarte, R. M., Barreto, R. M., Magalhães, J. P., Bastos, C. C. M., Ren, T. I., & Cavalcanti, G. D. C. (2014). Enhanced real-time head pose estimation system for mobile device. *Integrated Computer-Aided Engineering*, *21*(3), 281–293. <https://doi.org/10.3233/ICA-140462> |
| Yaw | - mean_yaw - median_yaw - std_yaw - MAD_yaw | Yaw head pose captures head side to side movements. Yaw was calculated as the differences in nose x coordinate between consecutive frames. | Arcoverde Neto, E. N., Duarte, R. M., Barreto, R. M., Magalhães, J. P., Bastos, C. C. M., Ren, T. I., & Cavalcanti, G. D. C. (2014). Enhanced real-time head pose estimation system for mobile device. *Integrated Computer-Aided Engineering*, *21*(3), 281–293. <https://doi.org/10.3233/ICA-140462> |
| Nose Aspect Ratio | - mean_NAR - median_NAR - std_NAR - MAD_NAR | **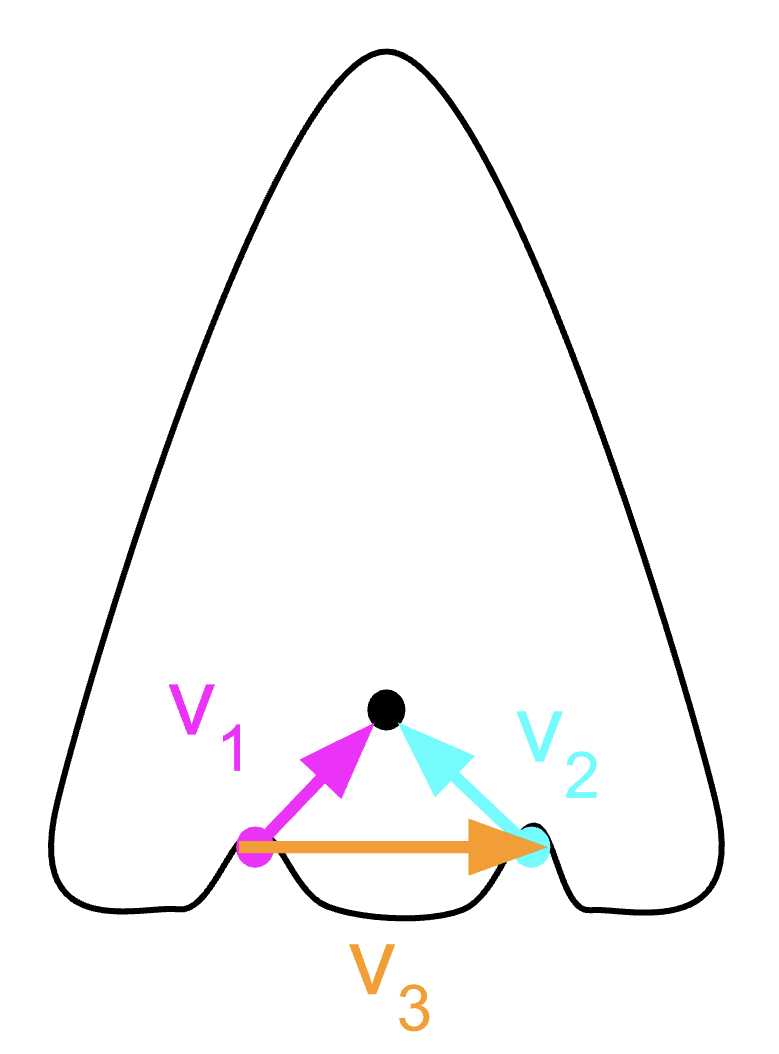**  Nose aspect ratio was another parameters used to represent in-plane head rotation. It was defined as  $\frac{v_{1}+v_{2}}{2*{\parallel v}_{3}\parallel}$ |  |
| Mean right thumb displacement, mean left thumb displacement, average mean thumb displacement, median right thumb displacement, median left thumb displacement, average median thumb displacement, right thumb displacement standard deviation, left thumb displacement standard deviation, etc. | - mean_thumbR - mean_thumbL - avg_mean_thumb - median_thumbR - median_thumbL - avg_median_thumb - std_thumbR - std_thumbL - avg_std_thumb - MAD_thumbR - MAD_thumbL - avg_MAD_thumb | Thumb displacement was calculated frame-to-frame by computing the Euclidean distance between consecutive (x,y) coordinates of the tip of the thumb |  |
| Mean right hand displacement, mean left hand displacement, average hand displacement, median right hand displacement, median left hand displacement, average median hand displacement, right hand displacement standard deviation, left hand displacement standard deviation, etc. | - mean_handR - mean_handL - avg_mean_hand - median_handR - median_handL - avg_median_hand - std_handR - std_handL - avg_std_hand - MAD_handR - MAD_handL - avg_MAD_hand | Hand displacement was calculated frame-to-frame by computing the Euclidean distance between consecutive (x,y) coordinates of the center of each hand |  |
| Mean right wrist displacement, mean left wrist displacement, average wrist displacement, median right wrist displacement, median left wrist displacement, average median wrist displacement, right wrist displacement standard deviation, left wrist displacement standard deviation, etc. | - mean_wristR - mean_wristL - avg_mean_wrist - median_wristR - median_wristL - avg_median_wrist - std_wristR - std_wristL - avg_std_wrist - MAD_wristR - MAD_wristL - avg_MAD_wrist | Wrist displacement was calculated frame-to-frame by computing the Euclidean distance between consecutive (x,y) coordinates of the point of the wrist that articulates with the radius |  |
| Mean right elbow displacement, mean left elbow displacement, average elbow displacement, median right elbow displacement, median left elbow displacement, average median elbow displacement, right elbow displacement standard deviation, left elbow displacement standard deviation, etc. | - mean_elbowR - mean_elbowL - avg_mean_elbow - median_elbowR - median_elbowL - avg_median_elbow - std_elbowR - std_elbowL - avg_std_elbow - MAD_elbowR - MAD_elbowL - avg_MAD_elbow | Elbow displacement was calculated frame-to-frame by computing the Euclidean distance between consecutive (x,y) coordinates of the tip of the elbow |  |
| Shoulder R-Shoulder L-Torso Triangle (STT or chest openness) | - mean_STT - median_STT - std_STT - MAD_STT | $\frac{\left\vert\left\vert Torso-Shoulder_{R} \right\vert\right\vert+\left\vert\left\vert Torso-Shoulder_{L} \right\vert\right\vert}{2\cdot\left\vert\left\vert Shoulder_{R} - Shoulder_{L} \right\vert\right\vert}$  The euclidean distance between each shoulder and torso was calculated for each frame and normalized by dividing two times the shoulder width. This metric represents the relative expansion of the upper body. Higher values indicate greater chest openess. | 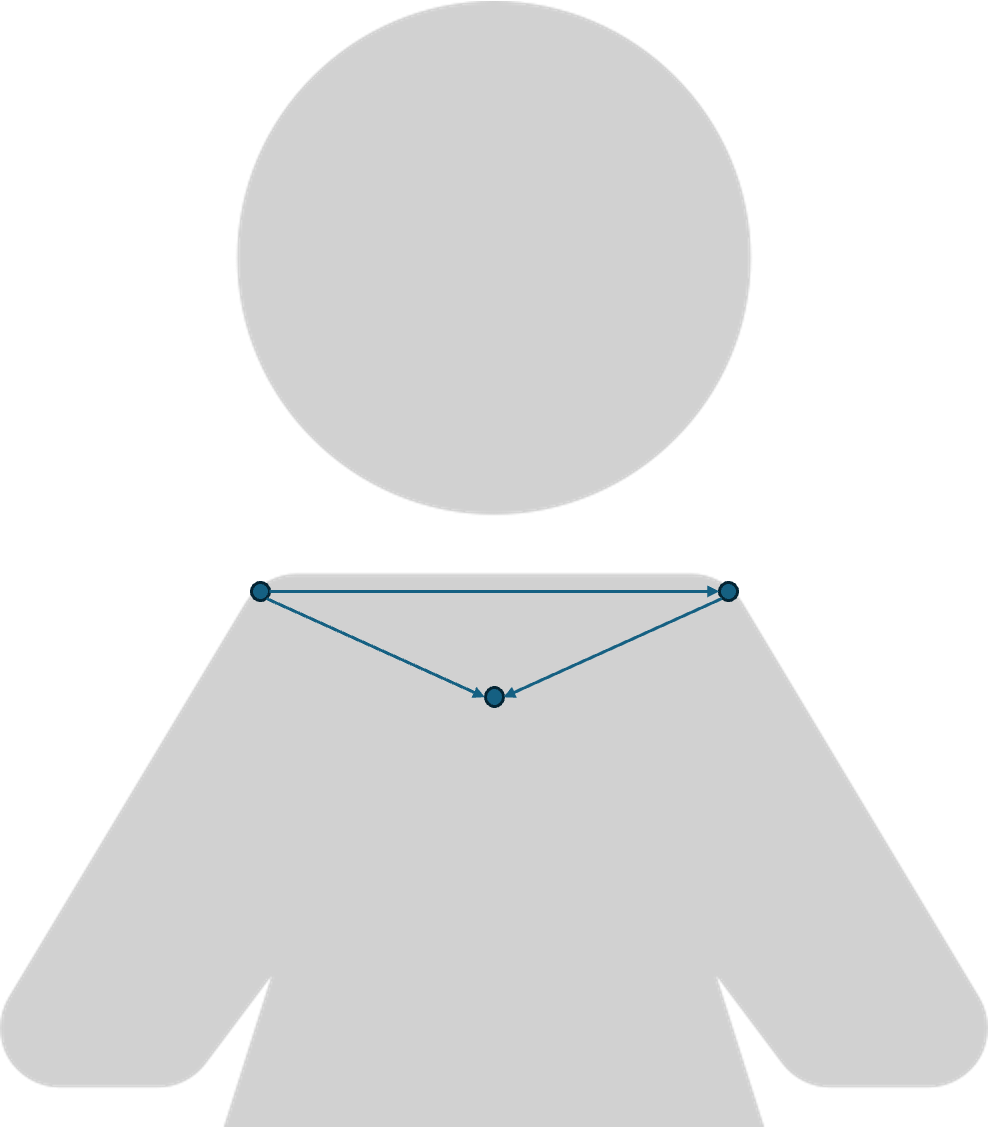 |
| Shoulder-Wrist-Elbow Angle (SWE) | - mean_sweR - mean_swe - avg_mean_swe - median_sweR - median_sweL - avg_median_swe - std_sweR - std_sweL - avg_std_swe - MAD_sweR - MAD_sweL - avg_MAD_swe | The shoulder-wrist-elbow angle is calculated for each frame by computing the arctangent of the ratio between shoulder-elbow length (upper arm) and elbow-wrist (forearm) length. It approximates changes in arm configuration. Note that this does not represent a true anatomical joint angle. | 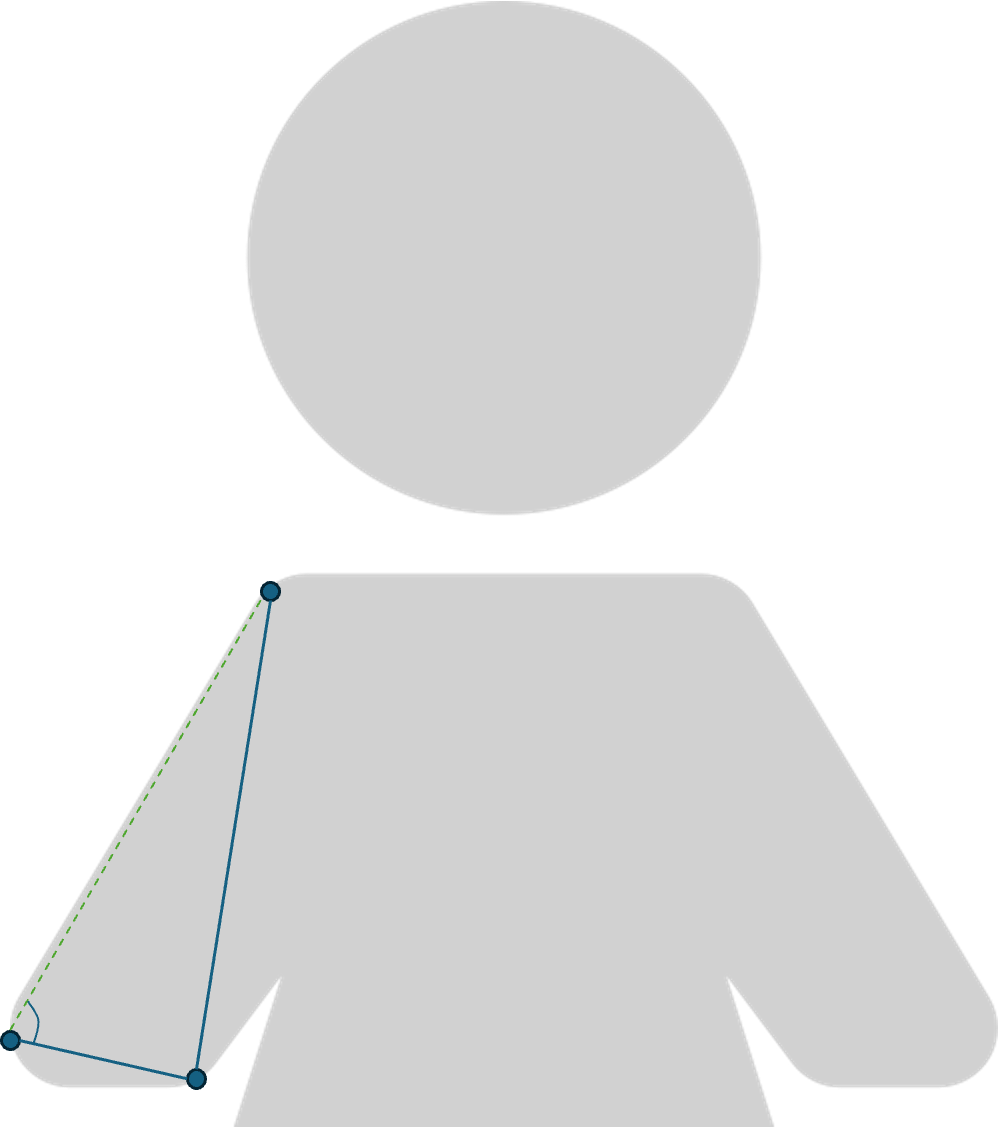 |
| Wrist-Shoulder-Elbow Angle (WSE) | mean_wseR, mean_wseL, avg_mean_wse, median_wseR, median_wseL, avg_median_wse, std_wseR, std_wseL, avg_std_wse, MAD_wseR, MAD_wseL, avg_MAD_wse | The wrist-shoulder-elbow angle is calculated for each frame by computing the arctangent of the ratio between the elbow-wrist length (forearm) and shoulder-elbow length (upper arm). Note that this does not represent a true anatomical joint angle. | 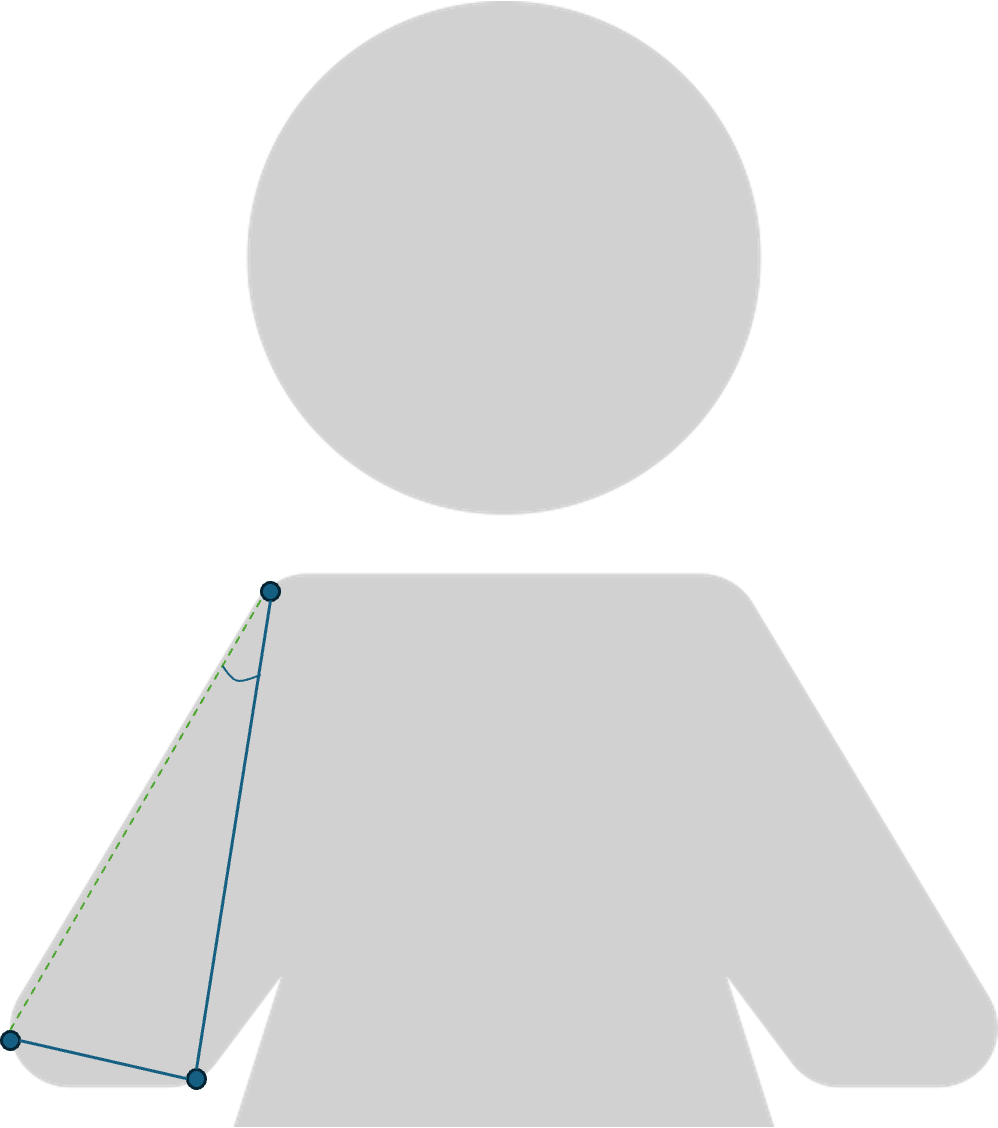 |
| Thumb-Wrist-Hand Angle (TWH) | mean_twhR, mean_twhL, avg_mean_twh, median_twhR, median_twhL, avg_median_twh, std_twhR, std_twhL, avg_std_twh, MAD_twhR, MAD_twhL, avg_MAD_twh | The thumb-wrist-hand angle is calculated for each frame by computing the arctangent of the ratio betwen the thumb-to-hand length and wrist-to-hand length. It approximates changes in thumb configuration relative to the hand segment. Note that this does not represent a true anatomical joint angle. | 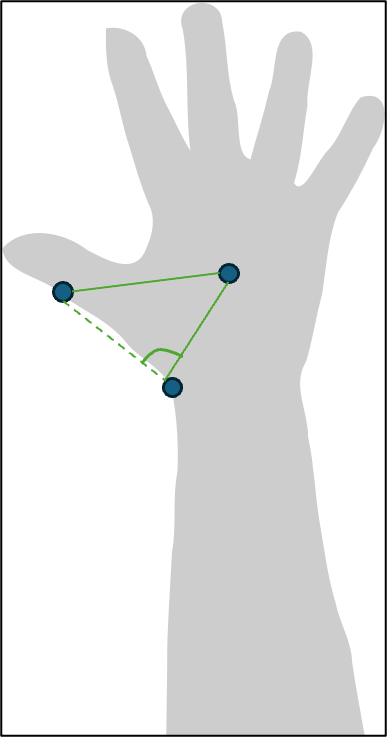 |
| Knee displacement | mean_kneeR, mean_kneeL, avg_mean_knee, median_kneeR, median_kneeL, avg_median_knee, std_kneeR, std_kneeL, avg_std_knee, MAD_kneeR, MAD_kneeL, avg_MAD_knee | Knee displacement was calculated frame-to-frame by computing the Euclidean distance between consecutive (x,y) coordinates of the knee near the patella |  |
