# Supplementary material for: Video-based detection of Delirium in hospitalized adults
Source: PLOS Digit Health. 2026 May 29;5(5):e0001462. doi: 10.1371/journal.pdig.0001462 (PMC13221075; doi:10.1371/journal.pdig.0001462)
Supplement: S5 Table — Model Hyperparameters. (DOCX) [file pdig.0001462.s011.docx]

| **Model** | **Hyperparameters** |
| --- | --- |
| SVM | - 'class_weight': 'balanced' - 'kernel': 'sigmoid' |
| Logistic Regression | - 'class_weight': 'balanced' - 'penalty': 'elasticnet' - 'solver': 'saga' |
| Gradient Boosting (XGB) | - 'objective' = 'binary:logistic' - 'max_depth': 2 |
| Random Forests | - 'class_weight': 'balanced' - 'max_depth': 3 |
