# Supplementary material for: Video-based detection of Delirium in hospitalized adults
Source: PLOS Digit Health. 2026 May 29;5(5):e0001462. doi: 10.1371/journal.pdig.0001462 (PMC13221075; doi:10.1371/journal.pdig.0001462)
Supplement: S6 Table — Keypoint Sensitivity and Specificity by Algorithm, Body Region, and Dataset. Sensitivity and specificity of different keypoint identification models based on the body region (face or extremity) and dataset (restricted or comprehensive). Only non-training images were used to calculate these statistics. Given the clustered nature of the data, a sandwich estimator of the variance was used to calculate 95% confidence intervals, using a logistic regression model with clustering by image (see Methods). (DOCX) [file pdig.0001462.s012.docx]

|  |  | **Restricted Dataset** | | **Comprehensive Dataset** | |
| --- | --- | --- | --- | --- | --- |
| **Model** | **Region** | **Sensitivity** | **Specificity** | **Sensitivity** | **Specificity** |
| DeepLabCut | Face | 0.90  [0.88, 0.92] | 0.77  [0.71, 0.82] | 0.97  [0.95, 0.99] | 0.74  [0.69, 0.78] |
| FaceMesh | Face | 0.78  [0.69, 0.85] | 0.39  [0.26, 0.54] | 0.74  [0.60, 0.84] | 0.71  [0.61, 0.79] |
| DeepLabCut | Extremity | 0.39  [0.32, 0.46] | 0.96  [0.95, 0.98] | 0.82  [0.78, 0.86] | 0.97  [0.94, 0.98] |
| BlazePose | Extremity | 0.76  [0.70, 0.82] | 0.47  [0.36, 0.59] | 0.78  [0.70, 0.84] | 0.43  [0.28, 0.59] |
