# Supplementary material for: Video-based detection of Delirium in hospitalized adults
Source: PLOS Digit Health. 2026 May 29;5(5):e0001462. doi: 10.1371/journal.pdig.0001462 (PMC13221075; doi:10.1371/journal.pdig.0001462)
Supplement: S7 Table — Keypoint Sensitivity and Specificity of the DeepLabCut Model by Gender and Body Region. Sensitivity and specificity of the comprehensive DeepLabCut model based on gender (female or male) and body region (face or extremity). (DOCX) [file pdig.0001462.s013.docx]

| **Region** | **Gender** | **Sensitivity** | **Specificity** |
| --- | --- | --- | --- |
| Extremity | F | 0.796 (293/368) | 0.978 (354/362) |
| Extremity | M | 0.844 (442/524) | 0.956 (302/316) |
| Face | F | 0.990 (1418/1432) | 0.770 (134/174) |
| Face | M | 0.961 (1595/1659) | 0.709 (134/189) |
