# Supplementary material for: Video-based detection of Delirium in hospitalized adults
Source: PLOS Digit Health. 2026 May 29;5(5):e0001462. doi: 10.1371/journal.pdig.0001462 (PMC13221075; doi:10.1371/journal.pdig.0001462)
Supplement: S9 Table — Univariate Behavioral Feature Differences. Exploratory univariate comparisons of video-based behavioral features significantly different between patients with or without delirium are shown in the table. Statistical significance was defined to be a p-value below 0.05 and was calculated using the Mann Whitney U-Test, which compared the median feature values between patients with or without delirium. P-values were not corrected for multiple comparisons given the exploratory and descriptive nature of this presentation. (DOCX) [file pdig.0001462.s015.docx]

|  | **Patients with Delirium** | | **Patients without Delirium** | |  |
| --- | --- | --- | --- | --- | --- |
| **Feature Name** | **Median** | **IQR** | **Median** | **IQR** | ***P*** |
| % Eyes Open | 95.11 | 16.79 | 99.68 | 1.93 | 0.029 |
| % Right Eye Open | 93.95 | 29.39 | 98.41 | 9.71 | 0.045 |
| % Left Eye Open | 85.91 | 65.92 | 98.04 | 11.27 | 0.026 |
| Mean Upper Lip Movement | 0.39 | 0.13 | 0.52 | 0.27 | 0.005 |
| Average Mouth Movement | 0.45 | 0.15 | 0.58 | 0.29 | 0.042 |
| Upper Lip Movement Median Absolute Deviation | 0.13 | 0.05 | 0.17 | 0.09 | 0.027 |
| Mean Right Pupil Displacement | 0.36 | 0.15 | 0.47 | 0.29 | 0.020 |
| Mean Left Pupil Displacement | 0.38 | 0.17 | 0.48 | 0.27 | 0.010 |
| Average Mean Pupil Displacement | 0.36 | 0.15 | 0.48 | 0.26 | 0.012 |
| Right Pupil Displacement Median Absolute Deviation | 0.12 | 0.06 | 0.15 | 0.09 | 0.029 |
| Left Pupil Displacement Median Absolute Deviation | 0.12 | 0.07 | 0.16 | 0.09 | 0.010 |
| Average Pupil Displacement Median Absolute Deviation | 0.12 | 0.06 | 0.15 | 0.09 | 0.014 |
| Median Left Horizontal Eyelid Axis | -7.13 | 3.81 | -8.32 | 2.85 | 0.018 |
| Vertical Mouth Axis Standard Deviation | 2.78 | 2.21 | 2.03 | 1.24 | 0.041 |
| Vertical Mouth Axis Median Absolute Deviation | 1.66 | 1.37 | 1.24 | 0.74 | 0.020 |
| Average Mouth Distance Median Absolute Deviation | 1.81 | 1.07 | 1.35 | 0.69 | 0.016 |
| Left Blink Rate Standard Deviation | 18.08 | 76.99 | 4.94 | 12.97 | 0.23 |
| Head Pitch Standard Deviation | 0.37 | 0.14 | 0.47 | 0.28 | 0.009 |
| Head Yaw Standard Deviation | 0.42 | 0.15 | 0.59 | 0.40 | 0.002 |
| Mean Right Wrist Displacement | 2.28 | 25.04 | 1.04 | 2.35 | 0.032 |
| Median Chest Openness Area | 0.60 | 0.12 | 0.54 | 0.07 | 0.013 |
| Chest Openness Area Median Absolute Deviation | 0.01 | 0.01 | 0.00 | 0.01 | 0.014 |
| Median Left Wrist-Shoulder-Elbow Angle | 0.17 | 0.72 | 0.62 | 0.73 | 0.044 |
