# Supplementary material for: Video-based detection of Delirium in hospitalized adults
Source: PLOS Digit Health. 2026 May 29;5(5):e0001462. doi: 10.1371/journal.pdig.0001462 (PMC13221075; doi:10.1371/journal.pdig.0001462)
Supplement: S10 Table — Results of Different Classification Model and Feature Selection Combinations. Algorithms include LR = Logistic Regression; RF = Random Forests; SVM = Support Vector Machine; XGB = XGBoost. Feature Selection methods include None: all features included, Chi square test, Lasso Regression, mRMR: minimal Redundancy, Maximal Relevance, and MI = Mutual Information. Additional Abbreviations: ROC AUC = Receiver Operating Characteristic Area Under the Curve. Std = Standard Deviation. Sens = Sensitivity. Spec = Specificity. The row highlighted in green (SVM, mRMR, 40), represents the best performing model. (DOCX) [file pdig.0001462.s016.docx]

| **Algorithm** | **Feature Selection** | **Number of Features** | **Mean ROC AUC** | **Std. ROC AUC** | **Mean Sens.** | **Std. Sens.** | **Mean Spec.** | **Std. Spec.** |
| --- | --- | --- | --- | --- | --- | --- | --- | --- |
| LR | None | 232 | 0.71 | 0.13 | 0.48 | 0.22 | 0.86 | 0.06 |
| LR | Chi^2^ | 10 | 0.66 | 0.15 | 0.82 | 0.14 | 0.25 | 0.14 |
| LR | Chi^2^ | 20 | 0.66 | 0.16 | 0.78 | 0.20 | 0.39 | 0.15 |
| LR | Chi^2^ | 40 | 0.65 | 0.15 | 0.61 | 0.24 | 0.62 | 0.19 |
| LR | Chi^2^ | 80 | 0.68 | 0.12 | 0.51 | 0.20 | 0.82 | 0.08 |
| LR | Lasso | Variable | 0.71 | 0.12 | 0.85 | 0.15 | 0.26 | 0.13 |
| LR | mRMR | 10 | 0.73 | 0.12 | 0.67 | 0.24 | 0.65 | 0.22 |
| LR | mRMR | 20 | 0.74 | 0.11 | 0.60 | 0.18 | 0.81 | 0.09 |
| LR | mRMR | 40 | 0.76 | 0.10 | 0.60 | 0.20 | 0.84 | 0.06 |
| LR | mRMR | 80 | 0.72 | 0.12 | 0.55 | 0.20 | 0.84 | 0.06 |
| LR | MI | 10 | 0.56 | 0.13 | 0.37 | 0.24 | 0.70 | 0.24 |
| LR | MI | 20 | 0.55 | 0.13 | 0.36 | 0.22 | 0.76 | 0.18 |
| LR | MI | 40 | 0.59 | 0.13 | 0.38 | 0.20 | 0.80 | 0.08 |
| LR | MI | 80 | 0.64 | 0.13 | 0.45 | 0.20 | 0.83 | 0.08 |
|  |  |  |  |  |  |  |  |  |
| RF | None | 232 | 0.71 | 0.09 | 0.04 | 0.08 | 0.99 | 0.02 |
| RF | Chi^2^ | 10 | 0.64 | 0.15 | 0.14 | 0.14 | 0.95 | 0.04 |
| RF | Chi^2^ | 20 | 0.64 | 0.14 | 0.09 | 0.10 | 0.96 | 0.04 |
| RF | Chi^2^ | 40 | 0.69 | 0.09 | 0.08 | 0.09 | 0.97 | 0.03 |
| RF | Chi^2^ | 80 | 0.70 | 0.11 | 0.06 | 0.08 | 0.98 | 0.03 |
| RF | Lasso | Variable | 0.72 | 0.11 | 0.09 | 0.10 | 0.96 | 0.04 |
| RF | mRMR | 10 | 0.68 | 0.12 | 0.11 | 0.10 | 0.96 | 0.04 |
| RF | mRMR | 20 | 0.68 | 0.10 | 0.10 | 0.12 | 0.97 | 0.03 |
| RF | mRMR | 40 | 0.68 | 0.10 | 0.08 | 0.09 | 0.97 | 0.03 |
| RF | mRMR | 80 | 0.67 | 0.11 | 0.07 | 0.10 | 0.99 | 0.02 |
| RF | MI | 10 | 0.56 | 0.11 | 0.05 | 0.08 | 0.95 | 0.06 |
| RF | MI | 20 | 0.58 | 0.11 | 0.03 | 0.06 | 0.96 | 0.05 |
| RF | MI | 40 | 0.61 | 0.11 | 0.04 | 0.06 | 0.97 | 0.04 |
| RF | MI | 80 | 0.64 | 0.10 | 0.02 | 0.05 | 0.98 | 0.03 |
|  |  |  |  |  |  |  |  |  |
| SVM | None | 232 | 0.79 | 0.09 | 0.71 | 0.16 | 0.78 | 0.07 |
| SVM | Chi^2^ | 10 | 0.50 | 0.18 | 0.55 | 0.22 | 0.60 | 0.12 |
| SVM | Chi^2^ | 20 | 0.57 | 0.19 | 0.65 | 0.20 | 0.62 | 0.11 |
| SVM | Chi^2^ | 40 | 0.62 | 0.16 | 0.65 | 0.18 | 0.63 | 0.12 |
| SVM | Chi^2^ | 80 | 0.71 | 0.10 | 0.70 | 0.17 | 0.67 | 0.11 |
| SVM | Lasso | Variable | 0.61 | 0.14 | 0.62 | 0.21 | 0.63 | 0.11 |
| SVM | mRMR | 10 | 0.52 | 0.17 | 0.52 | 0.23 | 0.59 | 0.10 |
| SVM | mRMR | 20 | 0.62 | 0.16 | 0.63 | 0.23 | 0.65 | 0.11 |
| SVM | mRMR | 40 | 0.69 | 0.13 | 0.69 | 0.18 | 0.66 | 0.10 |
| SVM | mRMR | 80 | 0.73 | 0.09 | 0.76 | 0.14 | 0.67 | 0.11 |
| SVM | MI | 10 | 0.47 | 0.14 | 0.39 | 0.26 | 0.63 | 0.27 |
| SVM | MI | 20 | 0.53 | 0.15 | 0.41 | 0.24 | 0.66 | 0.20 |
| SVM | MI | 40 | 0.57 | 0.15 | 0.50 | 0.25 | 0.69 | 0.12 |
| SVM | MI | 80 | 0.68 | 0.12 | 0.61 | 0.20 | 0.74 | 0.10 |
|  |  |  |  |  |  |  |  |  |
| XGB | None | 232 | 0.67 | 0.11 | 0.12 | 0.14 | 0.95 | 0.05 |
| XGB | Chi^2^ | 10 | 0.64 | 0.14 | 0.16 | 0.14 | 0.92 | 0.06 |
| XGB | Chi^2^ | 20 | 0.62 | 0.12 | 0.13 | 0.13 | 0.92 | 0.06 |
| XGB | Chi^2^ | 40 | 0.61 | 0.10 | 0.15 | 0.13 | 0.92 | 0.05 |
| XGB | Chi^2^ | 80 | 0.62 | 0.12 | 0.16 | 0.12 | 0.92 | 0.07 |
| XGB | Lasso | Variable | 0.69 | 0.09 | 0.18 | 0.15 | 0.91 | 0.06 |
| XGB | mRMR | 10 | 0.63 | 0.12 | 0.18 | 0.14 | 0.91 | 0.07 |
| XGB | mRMR | 20 | 0.61 | 0.11 | 0.19 | 0.14 | 0.92 | 0.06 |
| XGB | mRMR | 40 | 0.61 | 0.13 | 0.17 | 0.13 | 0.91 | 0.06 |
| XGB | mRMR | 80 | 0.60 | 0.12 | 0.16 | 0.13 | 0.93 | 0.06 |
| XGB | MI | 10 | 0.59 | 0.13 | 0.15 | 0.14 | 0.90 | 0.07 |
| XGB | MI | 20 | 0.61 | 0.13 | 0.12 | 0.12 | 0.92 | 0.06 |
| XGB | MI | 40 | 0.64 | 0.12 | 0.15 | 0.14 | 0.93 | 0.06 |
| XGB | MI | 80 | 0.65 | 0.10 | 0.13 | 0.12 | 0.94 | 0.05 |
