# Supplementary material for: Video-based detection of Delirium in hospitalized adults
Source: PLOS Digit Health. 2026 May 29;5(5):e0001462. doi: 10.1371/journal.pdig.0001462 (PMC13221075; doi:10.1371/journal.pdig.0001462)
Supplement: Supplementary file 14 [file pdig.0001462.s007.docx]

| Body Parts | Description |
| --- | --- |
| PupilR | pupil |
| PupilL | pupil |
| EyelidRtop | eyelid top; outline of eyelid closest to sclera |
| EyelidRmed | eyelid medial (closest to midline), outline of eyelid closest to sclera |
| EyelidRbot | eyelid bottom, outline of eyelid closest to sclera |
| EyelidRlat | eyelid lateral (away from midline), outline of eyelid closest to sclera |
| EyelidLtop | eyelid top, outline of eyelid closest to sclera |
| EyelidLmed | eyelid medial (closest to midline), outline of eyelid closest to sclera |
| EyelidLbot | eyelid bottom, outline of eyelid closest to sclera |
| EyelidLlat | eyelid lateral (away from midline), outline of eyelid closest to sclera |
| Forehead | glabella (folds of the forehead), above nasion |
| Nasion | indentation between forehead and nose where glasses rest |
| Nose | tip of nose |
| NostrilR | middle of the nostril (shadow) |
| NostrilL | middle of the nostril (shadow) |
| MouthTop | upper part of lip that borders the mouth |
| MouthR | right edge of lip |
| MouthBot | bottom part lip that borders the mouth |
| MouthL | left edge of lip |
| Chin | bottom of chin (boundary between face and neck) |
| EarR | dark opening of the ear canal |
| EarL | dark opening of the ear canal |
| KneeR | patella |
| KneeL | patella |
| ThumbR | tip of the first knuckle (which is the curve closest to the nail body) |
| ThumbL | tip of the first knuckle (which is the curve closest to the nail body) |
| WristR | the point of articulation of the wrist (curve) on the radial side (next to the thumb) |
| WristL | the point of articulation of the wrist (curve) on the radial side (next to the thumb) |
| HandR | center of hand either palm/frontal or dorsal facing |
| HandL | center of hand either palm/frontal or dorsal facing |
| ElbowR | tip of elbow (curve near olecranon) |
| ElbowL | tip of elbow (curve near olecranon) |
| ShoulderR | the curve of the shoulder closest to the neck |
| ShoulderL | the curve of the shoulder closest to the neck |
| Torso | sternum (manubrium - broad base) |
